# A development-centric perspective on pace-of-life syndromes

**DOI:** 10.1093/evlett/qrae069

**Published:** 2024-12-26

**Authors:** Isabel M Smallegange, Anja Guenther

**Affiliations:** School of Natural and Environmental Sciences, Newcastle University, Newcastle upon Tyne, United Kingdom; Research Group Behavioural Ecology of Individual Differences, Max Planck Institute for Evolutionary Biology, Plön, Germany

**Keywords:** energy allocation, energy acquisition, trade-offs, life-history theory, density-dependence

## Abstract

Organism responses to environmental change require coordinated changes across correlated traits, so-called syndromes. For example, animals differ in their “pace-of-life syndrome” (POLS); suites of correlated life-history, behavioral and physiological traits. But standard “gene-centric” evolutionary theory cannot explain why POLSs exist because it assumes that the expression of phenotypic traits of animals is determined by genotype-specified reaction norms; it ignores that developmental processes can bias the direction of evolution so that phenotypes no longer match genotype-by-environment interactions. Here we apply a development-centric perspective to derive new POLS hypotheses that can resolve the conflict that current POLS predictions fail to explain which species/populations are resilient to environmental change.

## Teaser

The gene-centric and development-centric perspective on pace-of-life syndromes (POLSs) differ in their assumptions on what trade-offs and mechanisms underlie and produce a POLS. Our focus on developmental systems motivates a conceptual change in pace-of-life syndrome studies that produces new predictions on POLS characteristics and population sensitivities to environmental change. In contrast to the classic model predictions, this development-centric perspective predicts fast types to be more responsive to environmental change because they have a larger energy budget to invest in flexibility or dispersal. The development-centric perspective bridges evolutionary theory with developmental biology by incorporating predictive-adaptive response and developmental constraint models, and allows linking POLS with eco-evolutionary dynamics such as niche construction, niche choice and niche conformance.

## Pace-of-life syndromes: where do we stand?

Organisms are entities and their response to environmental change involves coordinated changes of many, potentially correlated, and traits (Forsman, 2015; Schlighting & Pigliucci, 1998). Efforts to understand and predict how organisms respond to anthropogenic change, including global climate change, are now focusing on *multi-trait syndromes* (see Glossary)—sets of co-varying phenotypic traits. Several syndromes exist, including the dispersal (Stevens et al., 2014) and island (Adler & Levins, 1994) syndrome, but also the well-known pace-of-life syndrome (POLS), comprising suites of correlated life-history, behavioral, and physiological traits emerging from different solutions to life-history trade-offs (Dammhahn et al., 2018). Understanding the different paces among organisms at species-, population-, and individual-level, and how these affect responses to environmental change, is a major challenge to biologists (Del Giudice, 2020; Galipaud & Kokko, 2020). This is because an organism’s life history—its survival, *development*, and reproduction—directly influences the ecological dynamics of its population (Dochtermann, 2023; Sæther et al., 2016). Consequently, individual phenotypes and their competition for resources impact population growth through density-dependence and broader species-level processes. Understanding POLSs at levels from individuals to species is urgent because theory suggests that slow populations and species are buffered from increased environmental variation (McDonald et al., 2017; Morris et al., 2011; Sæther et al., 2016). However, field observations show that slower species are often more threatened than faster ones (Cardillo et al., 2005; Carmona et al., 2021; Xiong et al., 2024). We thus need to reconcile these theoretical predictions with empirical findings to determine which populations or species are resilient to change.

**Figure 1.**
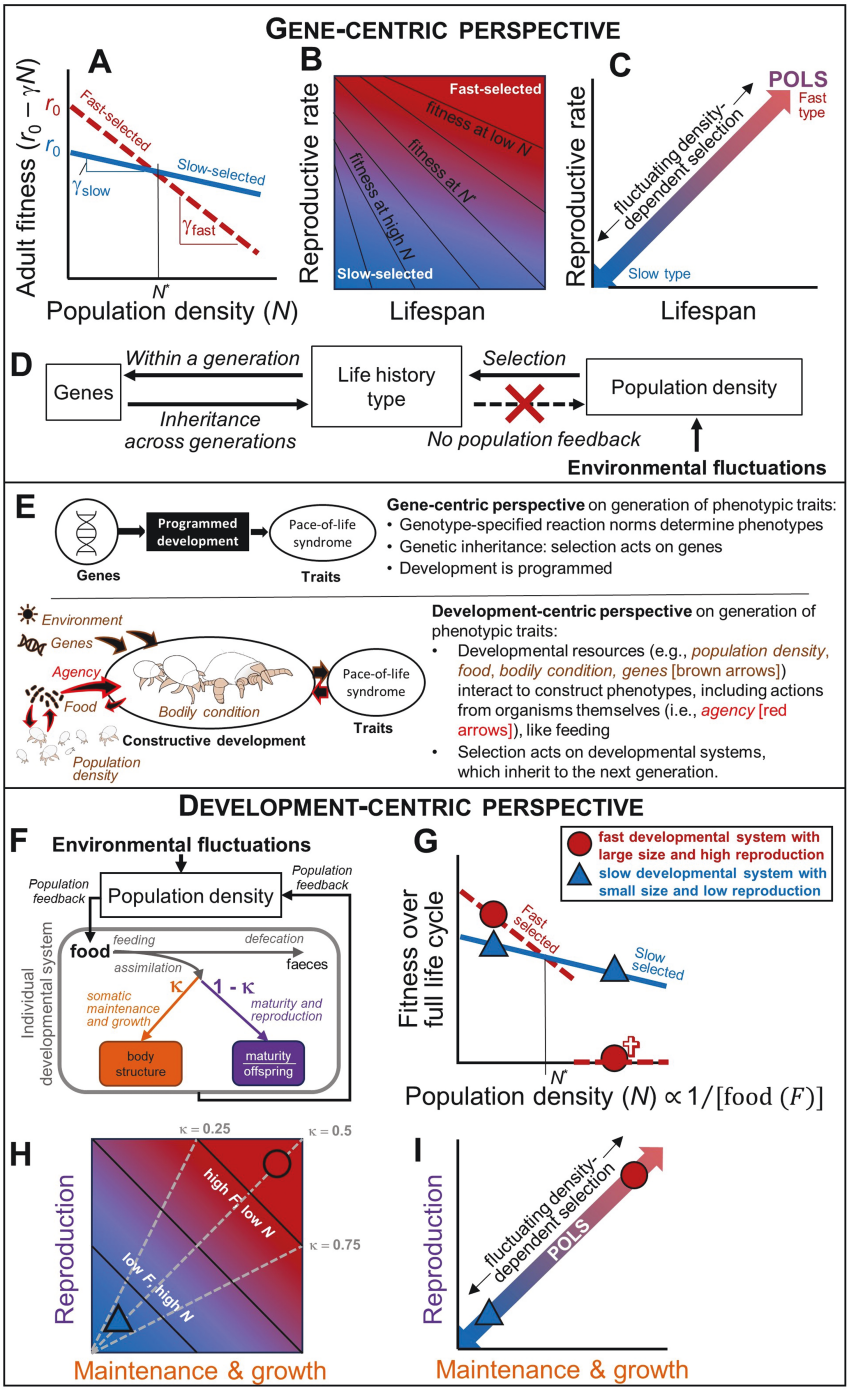
The gene-centric and development-centric perspective on the pace of life continuum in life history variation. (A) Gene-centric perspective (adapted from Wright et al., 2019)): faster genotypes have higher intrinsic reproduction (*r*_0_) than slower genotypes, but the lifespan of slower genotypes is reduced less by competition (γ) than that of faster genotypes. Thus, faster genotypes have higher fitness (*r*_0_ − γN) at lower population densities, and slower genotypes have higher fitness at higher population densities. (B) Plotting the negative trade-off between lifespan and reproductive rate across the fitness landscape highlights how fast-selected genotypes are favored at low population density and slow-selected genotypes at high population density. Note that at the intermediate population density N* (A), a pace-of-life exists where faster genotypes with low lifespan and high reproductive rate have the same fitness as slower genotypes with the opposite characteristics (along the antidiagonal in B). (C,D) Contrasting environmental fluctuations affect population density and create a fluctuating density-dependent selection that generates variation in fast versus slow genotypes, producing a pace-of-life syndrome: POLS (C: two-headed arrow). (E) Top: Gene-centric perspective as informed by standard evolutionary theory (Futuyma & Kirkpatrick, 2017). Bottom: development-centric perspective (Oyama et al., 2001). The bulb mite *Rhizoglyphus robini* was chosen for the illustration in (E) as it has been used as a model organism for questions framed in both perspectives (Smallegange et al., 2019) (Box 1). (F) Development-centric perspective: Individuals acquire energy from food, of which a fraction κ goes to maintenance and growth (in orange/left-hand arrow) and the rest to maturity and reproduction (in purple/right-hand arrow). (G) Food levels (*F*, which ranges from *F* = 0 [empty gut] to *F* = 1 [full gut]) are inversely related to population density. At high food levels, individuals grow to a large size and reproduce at a high rate so that fitness is high (red circles). But, these fast developmental systems starve at low food levels (i.e., high densities), because they cannot cover maintenance costs. Slower developmental systems (blue triangles) are smaller and have a lower reproduction rate but can cover maintenance costs at low feeding levels, and survive. At higher feeding levels, however, these slower systems have lower fitness than the faster ones (H). Plotting the negative trade-off between growth and reproduction across a range of food levels (*F*) highlights how fast-selected developmental systems are favored at low population density and slow-selected developmental systems at high population density. Note that at the same, constant food levels (black solid lines), individuals can achieve the same fitness by adopting different allocation strategies (κ: gray dashed lines). This produces a pace-of-life at constant population densities that are driven by energy allocation differences (rather than differences in energy acquisition across population densities that underlie the pace-of-life syndrome: G). (I) Contrasting environmental fluctuations affect population density and create a fluctuating density-dependent selection that generates variation in fast versus slow developmental systems, producing a pace-of-life syndrome: POLS (two-headed arrow).

The original POLS hypothesis proposed correlational selection as a potential mechanism for this functional integration of traits (Dammhahn et al., 2018). However, two meta-analyses have found no support for genetic correlations in line with POLS (Chang et al., 2024; Royauté et al., 2018). Researchers now recognize that environmental conditions shape POLS on shorter ecological rather than evolutionary time scales (Hämäläinen et al., 2021; Montiglio et al., 2018). A newly proposed conceptual model states that POLSs within and between populations arise due to variations in population density (Wright et al., 2019). This model of *fluctuating density-dependent selection* POLS is built upon classical *r*- and *K*-selection theory (MacArthur & Wilson, 1967; Pianka, 1970) and its extensions (Engen et al., 2013; Engen & Sæther, 2017; Lande et al., 2009; Milles et al., 2022). It is also supported by empirical data (Sæther et al., 2016) that suggest a trade-off between inherent rates of reproduction (*r*_0_; density independent) and the responsiveness to intraspecific competition (γ; density dependent) (Milles et al., 2022). Wright et al., (2019) explain that the trade-off between *r*_0_ and γ relates to the trade-off between current versus future reproduction and produces a pace of life axis along a gradient of fluctuating density dependence. Fast-reproducing genotypes thrive in low-density populations, while slow-reproducing genotypes that can withstand competition are favored when density increases (Figure 1A). Population density variations, caused by regular (seasonal) or random environmental effects, drive density-dependent selection, which can lead to the evolution and maintenance of POLS within and among populations (Wright et al., 2019) (Figure 1B–D). Engen et al., (2020) expanded theory for environmentally induced stochastic selection by including how much an individual with a particular phenotype contributes to within-species competition for resources. This approach mimicked density regulation through resource use rather than just population size, but still showing that species can be classified along a slow-fast pace-of-life continuum (Engen et al., 2020).

The conceptual models developed by Wright et al., (2019; 2020) are characteristic of how POLS models take a *gene-centric perspective*, because they assume covariation between life history and behavior by genetic correlations (Montiglio et al., 2018; Vasilieva et al., 2022) (Table 1), even though support for such genetic correlations is lacking (Chang et al., 2024; Hämäläinen et al., 2021; Prahb et al., 2023; Royauté et al., 2018). Environmental influences on phenotype expression can be identified using variance partitioning methods that distinguish between genetic versus permanent environmental (including what Wright et al., (2019) call developmental plasticity) or current environmental causes of phenotypic variation. Even so, this quantitative genetic approach does not incorporate processes but only specifies outcomes of development as a range of phenotypes possible from the genotype of an individual. The lack of mechanistic underpinnings to the process (instead of the outcome) of phenotype development makes it fundamentally impossible to accurately forecast population responses to novel conditions (Mathot & Frankenhuis, 2018; Smallegange, 2022), e.g., those created by climate change. An alternative, “development-centric” perspective can help create a predictive framework of the pace-of-life (Oyama et al., 2001; Prahb et al., 2023) (Figure 1E) (Table 1).

**Table 1.** The assumptions and predictions of state-of-the-art eco-evolutionary models by Wright et al., (2019) (gene-centric perspective) and proposed here (development-centric perspective (Oyama et al., 2001; Smallegange, 2022) to explain the pace-of-life syndromes (POLSs).

	*Gene-centric perspective*	*Development-centric perspective*
Assumptions		
Fast versus slow types	Fast types have high reproduction rates and perform best at low population densities, whereas slow types with high investment in competitive traits and longevity perform best at high densities, but at the cost of delayed reproduction.	Individuals at high(er) energy levels reach large(r) sizes and have high(er) reproduction, and are fast(er) types.
Inheritance	Genetic inheritance and selection acts on genes.	Selection acts on developmental systems, which are inherited by the next generation.
Life history co-variation	Due to genetic correlations.	Due to differences in energy budget and energy requirements.
Influence of the environment on phenotype expression	A range of phenotypes is possible from the genotype of an individual in relation to permanent and current environmental conditions (genotype-specified reaction norms).	Environmental conditions and agency affect development, transforming phenotypes and altering the response to selection.
Resource acquisition and allocation	Slower types implicitly are assumed to have fewer resources available because they always allocate resources to competitive ability.	Explicitly included through κ and energy acquisition. Acquisition is inversely related to population density due to competition over energy.
Population feedback	Neither fast nor slow types influence population density (no feedback to population ecology).	Feedback to population ecology through energy acquisition (density-dependence).
***Predictions***		
POLS	A fast–slow axis of variation in life history, physiological, morphological, and behavioral traits with highly fecund, short-lived, and dispersive “fast” types at one end and less fecund, long-lived, competitive, and philopatric “slow” types at the other, aligns with fluctuations in density-dependent selection within or among populations.	Fast, large types with high fitness develop at low density but with high resource acquisition. At high density, these types suffer due to high maintenance costs (Figure 1G). Slow types avoid impairment at high densities by lower maintenance costs, yet they suffer from reduced growth and reproduction (Figure 1G). Fluctuating density-dependent selection produces a fast–slow axis of variation in developmental systems and linked traits.
Sensitivity to environmental change	Slower types are more responsive because their higher survival rate (due to high investment into competitive traits) is assumed to be selected for increased investment into plasticity and dispersal.	Faster types are more responsive because they have a higher energy budget that they can invest in plasticity or dispersal. However, if the environmental change is a drastic, persistent reduction in food, only the slow types will survive because they have low maintenance costs, whereas fast types will starve.

A recent *development-centric perspective* on the evolution of phenotypes (Oyama et al., 2001) (Fig. 1E: bottom) is still contentious because it does not neatly slot into standard evolutionary theory (Uller & Laland, 2019; Uller et al., 2018). Yet, it offers an alternative conceptual framework to achieve an in-depth understanding of unexplained biological phenomena. For example, Araya‐Ajoy et al. (2021) found that house sparrows (*Passer domesticus*) reproduce at an older age in high-density conditions and at a younger age in low-density conditions. On the one hand, this supports the classic density-dependent selection theory, where there is a trade-off between current and future reproduction (Araya‐Ajoy et al., 2021; Wright et al., 2020). But these findings can also be due to slower development under high density because of limited energy resources. It is also interesting that most variation in reproduction age was plastic (Araya‐Ajoy et al., 2021), indicating a strong developmental influence.

Evolutionary biologists view development predominantly within a gene-centric perspective, focusing on developmental outcomes (West-Eberhard, 2003; Gilbert & Epel, 2009; for a detailed analysis, see Uller et al., 2024). However, developmental processes enable organisms to accommodate perturbations, influencing the distribution of life histories within a population. This means that development can alter the response to selection (e.g., Deere & Smallegange, 2023) and thus cannot be fully integrated into standard evolutionary theory, which sees variation in adult phenotypes as stochastic events arising during development. From a development-centric perspective, life histories are produced in a process involving complex interactions between the environment surrounding an individual, its bodily condition, genes, and an individual’s own impacts on their environment (*agency*: Figure 1E, bottom) (Oyama et al., 2001; Smallegange, 2022). Development is constructive instead of programmed (Fig. 1E). The unit of selection is the life cycle or developmental system (Oyama et al., 2001) and evolution is the differential reproduction of variant developmental systems. The start of a new life cycle entails the rebuilding of the different mechanisms that enable the developmental system to reproduce itself from relatively basic resources, with gene replication being just one part of the process (Oyama et al., 2001).

## A development-centric perspective on pace-of-life syndromes

During development, organisms vary in their energy acquisition and allocation between maintenance and growth versus maturation and reproduction as environmental conditions fluctuate (Marquet et al., 2014). The amount of energy an organism can spend depends on its available resources like food, territories, nests, etc., the resource competition it experiences, and to what extent acquisition is genetically or developmentally induced. Recent studies suggest that heterogeneity in resource acquisition versus *allocation* is responsible for the mixed empirical support of POLS hypotheses (Haave-Audet et al., 2022; Laskowsi et al., 2021; Prahb et al., 2023). For example, in *Onthopagus* dung beetles, well-fed males develop large horns (Snell-Rood et al., 2011), driven by the expression of one specific gene (*dsx*). Knock-out of this gene reduces both horn expression and nutrition-intake (Kijimoto et al., 2012). Thus, a development-centric approach that considers environmental conditions and resource variation can improve our understanding of density-dependent selection and multi-trait relationships (Prahb et al., 2023).

When resource availability directly translates into energy availability, a simple, energy budget model of energy allocation that assumes isomorphic growth, states that individuals allocate a fraction κ to somatic maintenance and growth, and the rest of the energy (1 − κ) to maturity (juveniles) or reproduction (adults) (Figure 1F) (Kooijman & Metz, 1984). At low density, acquisition is high and individuals grow quickly, reproduce more, and have high fitness. But, at high density, the high maintenance costs of these *fast-paced types* can lead to starvation and death (Figure 1G,H). In contrast, *slow-paced types* fare better at high densities due to lower maintenance costs (Kooijman & Metz, 1984), but their slower growth and reproduction lowers their total fitness (Fig. 1G). Environmental fluctuations and population feedback create density-dependent selection, favoring fast development at low density, and slow development at high density (Smallegange, 2022) (Figure 1F), producing a POLS across an energy acquisition gradient (Fig. 1I) (variation in energy allocation [κ] is assumed to be low). Note that if populations evolve different energy assimilation strategies in response to permanent environmental change (Cuthbert et al., 2016; Smallegange & Deere, 2014), this moves the POLS along the acquisition gradient. Note also that if variation in allocation is (much) greater than acquisition, the trade-off between growth and reproduction produces a pace of life that varies from fast types that invest in reproduction (low κ) to slow types that instead invest the same acquired energy into maintenance and growth (high κ) (Figure 1C: antidiagonal). This POLS is maintained, for example, by weak selection when fitness differences between the two types are small, like near the cross point of the fitness functions (Figure 1G). Importantly, this perspective includes population feedback through e.g., density-dependent competition over food (Figure 1F), unlike the gene-centric perspective.

## Assessing assumptions of the two perspectives

### Assumptions on energy acquisition and allocation

The gene-centric perspective assumes that slower genotypes always invest more energy into competitive ability (Luttbeg & Sih, 2010; Wright et al., 2019) (Table 1). Thus, even at low densities, they have less energy available than faster types, resulting in lower reproductive rates (*r*_0,slow_ < *r*_0,fast_: Figure 1A). At higher densities, however, they outperform faster genotypes, which have limited competitive ability (Figure 1A). Only at the population density where fitness functions cross do fast and slow genotypes have equal fitness (producing a pace of life that is aligned closely with variation in allocation between lifespan versus reproductive rate [Figure 1B: antidiagonal]). Together, this means that, from a gene-centric perspective, trait covariation along the POLS relates to variation in both acquisition and allocation, produced by fluctuating, density-dependent selection (Figure 1C).

Our model from a development-centric perspective explicitly links the pace of life to energy allocation at any given level of energy acquisition (Figure 1F). It assumes that the energy acquired from resources like food inversely relates to population density because of scramble or interference competition. Therefore, at high food levels, growth and reproduction are high (Figure 1H: red circle). However, these fast types starve at low food (energy) levels because they cannot cover their maintenance costs (Figure 1G) (Table 1). Slower types (blue triangles) (Figure 1G and H) have lower maintenance costs at the same allocation level, and can survive at low food levels (Figure 1G), but have lower growth and reproduction. Thus, if all individuals adopt the same energy allocation strategy (κ), the development-centric perspective predicts a POLS along an energy acquisition gradient, produced by fluctuating density-dependent selection on developmental systems with different energy budgets (Figure 1I).

In reality, individuals will alter their allocation strategy in response to energy acquisition. For example, at low energy acquisition, individuals who can pay their maintenance costs could prioritize growth over reproduction or vice versa at high energy acquisition levels. In fact, intraspecific variation in energy budget parameters can be substantial (Potter et al., 2021). Whereas the approach we outline here assumes that energy allocation to growth versus reproduction (κ) is fixed (Kooijman & Metz, 1984), this assumption can be relaxed. Thunell et al., (2023), e.g., varied energy allocation (κ) to reflect (for fish in general and pike [*Esox lucius*] specifically) that reproductive energy investment is increasingly prioritized over growth, as body size increases. Martin et al., (2012) even developed a generic individual-based model based on dynamic energy budget theory (Kooijman, 2010), that considers variation among individuals, local interactions, and adaptation in a rigorous manner. If individuals differ in their developmental system, including in e.g., their energy allocation, and if developmental systems reliably inherit to the next generation, selection can act on them and evolve developmental systems (Table 1; Figure 1E).

### Assumptions on population feedback can impact predictive power

Individual life histories and population density dynamics are interconnected, particularly through density-dependent competition for food (Smallegange & Deere, 2014). The resulting variation in survival, growth, and reproduction directly impacts ecological population dynamics (Smallegange et al., 2017), which in turn can affect the strength of density-dependent selection individuals experience (e.g., Smallegange & Deere, 2014). In this way, population feedback can transform the response to selection so that it is no longer straightforward to predict how selection impacts life histories. For example, in food-restricted, male dimorphic bulb mite populations (*R. robini*: Figure 1E) males evolved over 15 generations to forego the development of “fighter” legs (that can be used to kill conspecifics), regardless of which adult male morph was selected against (Smallegange & Deere, 2014). This result can only be explained if variation in population density and internal energy economies during development are more important evolutionary drivers of male morph development and life history than adult male performance alone (Smallegange et al., 2019). It is likely that selection on *r*_0_ and γ (Figure 1A) impacts population size and structure, but, crucially, population feedback can impact this response, as *r*_0_ and γ will depend on energy investment (see also next section). For example, if population feedback through e.g., feeding is so strong that individuals are unable to significantly invest in γ (competitive ability), any positive selection on γ will have little impact. This is because the strong population feedback under high-density dependence during development is a stronger evolutionary driver, selecting against investment into γ but into viability and essential physiological processes, like the aforementioned example (Smallegange & Deere, 2014). The gene-centric perspective on POLS does not include population feedback (Table 1; Figure 1D). In fact, changes in density-dependent selection are assumed to be caused by population densities being affected by environmental stochasticity (e.g., weather) or demographic stochasticity (e.g., predation) (Wright et al., 2019). More generally, the gene-centric perspective can include environmental influences on phenotype expression in genotype-specified reaction norms that capture genotype-by-environment interactions (Lynch & Walsh, 1998) (Table 1). Their relative contributions can be disentangled using variance partitioning methods, including environmental feedback effects through density-dependence (Coulson, 2021). However, such reaction norms give the (genotype-by-environment-specified) outcomes of development and do not accommodate developmental processes. A development-centric perspective on density-dependent selection instead offers a mechanistic basis to include population feedback in predictions on how a POL shapes population responses to novel change (Table 1; Figure 1F).

## Plasticity and predictions on responsiveness to environmental change

The development-centric perspective predicts that fast types are more responsive to environmental change because they have a higher energy budget and can thus invest in plasticity or dispersal in response to environmental change (Table 1). However, if the environmental change is a drastic, persistent reduction in food or other resources closely related to building and maintaining energy, they are disadvantaged due to their high maintenance costs (Kooijman & Metz, 1984). The gene-centric perspective, in contrast, predicts that slow genotypes are more responsive to environmental change because they are more phenotypically plastic than faster genotypes (Wright et al., 2019) (Table 1). Teasing apart which mechanism (trade-off between current and future reproduction of gene-centric POLS theory, or selection on the full life cycle of development-centric theory) drives POLS evolution thus starts by testing whether faster or slower individuals and populations are most sensitive to environmental change (Box 1) (e.g., Rademaker et al., 2024).

Box 1: **A practical guide to testing development-centric perspective POLS predictions**

The development-centric approach presented in this study is inspired by Dynamic Energy Budget (DEB) theory (Kooijman, 2010). DEB theory is a formal metabolic theory that provides a quantitative framework to describe how organisms uptake, store, and utilize energy and nutrients throughout their life cycle; it applies universally to all living animals, from bacteria to whales (Kooijman, 2010). DEB theory is incorporated into different categories of population models. One category is Physiologically Structured Population Models (PSPMs) (De Roos, 1997; Metz & Diekmann, 1986), which can be used to model the acquisition and use of energy for organisms during the entire life cycle to study density-dependent feedback effects between a population (or species) and its environment, and resulting patterns of life history evolution (de Roos & Persson, 2013). However, PSPMs are not straightforward to analyze, and typically are also more mathematical, rather than actual representations of biological systems (Smallegange et al., 2017). An alternative category of population models is Dynamic Energy Budget Integral Projection Models (DEB-IPM) (Smallegange et al., 2017; Thunell et al., 2023). DEB-IPMs are less mathematically complex and can be used to study population and life-history dynamics from an energy budget perspective, using data on survival, growth, and reproduction.

**Figure 2.**
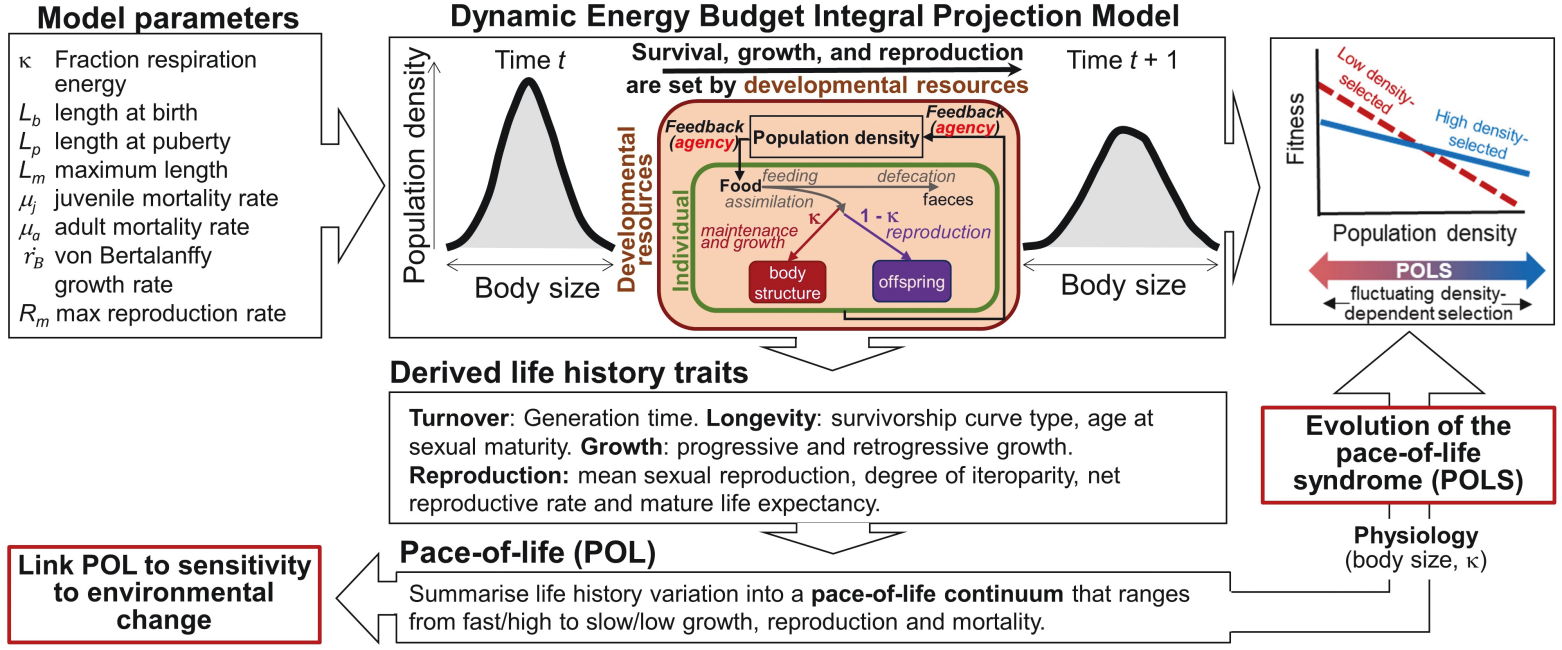
Schematic showing how a parameterized DEB-IPM (Smallegange et al., 2017) can be used for single or multiple ectotherm species to derive life history traits to calculate a pace-of-life (POL) axis (see Smallegange & Lucas [2024] for a “how-to” guide), and, when linked to e.g., physiological characteristics, to study the evolution of the pace-of-life syndrome (POLS).

One type of DEB-IPM (Smallegange et al., 2017; Figure 2: top) has been parameterized for >180 coldblooded species (Smallegange & Lucas, 2024). Details on how to estimate the eight model parameters (Figure 2 top left) for species or populations not yet included in the dataset are provided by Smallegange et al. [2017] and in Add-my-pet [2024]). One can parameterize DEB-IPMs for different species or for different populations of the same species. Once parameterized, one can calculate derived life history traits (listed in the middle of Figure 2) that inform on a population’s or species’ turnover rate, longevity, growth, and reproduction for different food levels and allocations to growth κ ([1 − κ] goes to reproduction). In this way, one can assess if this life history variation across populations or species can be structured into a pace-of-life (POL) continuum (Figure 2). By subsequently linking the POL to, e.g., physiological or behavioral characteristics one can identify if a pace-of-life syndrome (POLS) exists (Salguero-Gómez et al., 2016; Smallegange & Lucas, 2024) (Figure 2).

The most important difference between the gene- and development-centric POLS predictions is how populations respond to environmental change (Table 1): the gene-centric perspective states that slower types are more responsive to environmental change, whereas the development-centric perspective states the opposite. Using the above modeling approaches to identify if a POL(S) exists across populations or species, one can test this key prediction using elasticity analysis (Smallegange & Lucas, (2024) provide a how-to guide; Rademaker et al., (2024) provide an example for ray-finned fish). This key prediction can also be tested in laboratory or field experiments.

In laboratory experiments, one can for example evolve populations of the bulb mite *Rhizoglyphus robini* (Figure 1E), to adapt to low and high population densities. If, indeed, their characteristics (survival, growth, reproduction, dispersal, morphology) align along the POLS continuum (Table 1), one can subsequently expose them to environmental change. Warming, for example, impacts bulb mite survival, development, reproduction, and plasticity (Plesnar-Bielak et al., 2013; Rhebergen et al., 2022). The results will show if low and high-density-adapted populations respond differently to environmental change.

In field experiments, one can sample bulb mite populations in different vegetation sites in the field, to test if phenotypic characteristics change over a population density gradient. By subsequently moving the offspring into the laboratory, one can test if their response to this environmental change is explained by the POLS characteristics of their parents. One could also apply a warming treatment to each vegetation site and score real-life POLS dynamics under environmental change.

Slower genotypes are favored in populations with high-density dependent selection (Figure 1A and D) (Wright et al., 2019), which are presumed to exist in more predictable and less variable environments. Selection should thus favor investment into cue acquisition informative on future environmental conditions, fueling the evolution of *phenotypic plasticity* (Chevin & Lande, 2015; Wright et al., 2019). Perhaps this is why slower genotypes are assumed to have lower energy acquisition than faster genotypes (*r*_0,slow_ < *r*_0,fast_ [Figure 1A]) (in addition to energy investment into competitive ability, γ), otherwise it is unclear how they can invest into plasticity and competitive ability at the same time. In fact, Nicolaus et al. (2015) found that slower wild great tits (*Parus major*) produced clutches of optimal sizes, whereas faster types produced suboptimal clutch sizes. Nicolaus et al. (2015) speculate that, among others, faster birds were energetically constrained in their offspring production; again, this fails the implicit assumption of the gene-centric POLS model that faster genotypes have higher energy acquisition than slower ones (Figure 1A) (Wright et al., 2019). A more in-depth approach to the processes that underlie allocation into reproduction, as well as other processes, seems warranted if we are to understand links among plasticity, life history, and how individuals respond to environmental change (Box 2). For example, consistent individual differences in coping behaviors are assumed to mediate the allocation of limited energy to self-maintenance, reproduction, and other costly features such as staying plastic. Fast types are considered to have a “proactive” coping style, in which they do not track environmental changes but modify their reproduction based on internal routines; slower types have a more ‘reactive’ coping style and adaptively anticipate environmental conditions repeatedly (Coppens et al., 2010; Duckworth, 2006; Nicolaus et al., 2015). The field of developmental biology investigates the ontogeny and maintenance of such individual differences in behavior and life history in the context of several, partially contrasting hypotheses of *developmental plasticity* (Box 3).

Box 2: **Developmental systems and ecological niche-altering mechanisms**

Recent advances in ecology and evolution draw attention to the idea that individuals have their own individualized niches within populations due to ecological specializations (Takola & Schielzeth, 2022). Individuals interact with the environment in various ways, thereby altering population-level selection and evolution. Niche choice—individuals selecting a suitable habitat based on their own phenotype; niche conformance—individuals changing their phenotype to conform to a given environment and niche construction—individuals altering their environment according to their needs are currently hot-topics investigated in Ecology, Evolution, and Behavioral Biology (Gilbert et al., 2015; Trappes et al., 2022). These mechanisms often occur in concert. For example, Californian harvester ant queens differ in their tolerance toward other queens, and this leads, within the same population, to colonies that are built by just a single or multiple queens (Overson et al., 2016). These individual differences have been linked to resource availability in the environment, favoring one or another type of niche construction, and impacting the selective environment (Haney & Fewell, 2018). Applying a development-centric perspective could in this and other scenarios predict (rather than just observe the outcome in a correlative way) which environmental conditions should favor which nice construction pattern, thereby providing a predictive link to relevant ecological and evolutionary questions/ frameworks. Processes of niche construction have been demonstrated to influence evolutionary trajectories and are therefore expected to be involved in speciation and macroevolutionary shifts (Gilbert et al., 2015). For example, populations of *Pseudomonas fluorescens* evolved niche construction within ~100 generations when adapting to a novel environment in a laboratory experiment (Callahan et al., 2014). In current models, which are usually gene-centric, such niche construction is typically considered to arise due to varying selection across different environments while the distribution of genotypes is assumed to occur at random across different environments (Saltz, Nuzhdin & 2014).

Box 3: **Within-population POLSs and developmental plasticity**

The pace-of-life concept was originally developed to understand life history variation at the among-population level but has recently been extended to understand consistent individual differences in life history and behavior, i.e., animal personality, variation at the within-population level (Dammhahn et al., 2018; Réale et al., 2010). Early, gene-centric models predicted that fast and aggressive “bold” individuals who are poor explorers can coexist with slower, less bold but highly explorative individuals due to small, stochastic differences in distributed resources (Wolf et al., 2007). A recent meta-analysis however revealed an opposite relationship across taxa (Garamszegi et al., 2012). From a development-centric perspective, local environmental stochasticity can result in differences in energy acquisition and thus in individual conditions (Figure 1F), fueling variation in developmental systems among members of the same population. Such developmental differences can arise from the same genotype via changes in gene expression leading to continuous or discrete phenotypes; via changes in gene regulatory networks or via developmental switch genes (Gilbert et al., 2015).

Even if individuals experience the exact same conditions, within a population they can achieve the same fitness by adopting different energy allocation strategies (Fig. 1H: antidiagonal). Such alternative (reproductive) strategies are well described across the animal kingdom. While in some species, they are genetically determined, in others, they are the phenotypic expression of differentially expressed genes due to different environmental trajectories (Snell-Rood et al., 2011).

A crucial issue is the degree to which phenotypic plasticity is responsible for the existence of within-population POLSs (Wright et al., 2019). For instance, proactive and reactive coping styles can be seen as adaptive developmental reactions to various early-life experiences. Such shaping of phenotypes may come about via direct environmental effects or may be transmitted by parents to prepare offspring optimally for the environment they will face (Groothuis & Taborsky, 2015). Such different developmental trajectories can be understood in the context of predictive adaptive responses (PARs) (Bateson et al., 2014; Gluckmann et al., 2005a, 2005b). According to the external PAR theory, early-life adversity acts as a “weather forecast” for the environmental conditions that individuals will grow into (Ellis et al., 2009; Gluckman & Hanson, 2006). Therefore, it is beneficial for “reactive” individuals to respond by forming a reproductive plan suitable for the expected environment. The internal PAR theory suggests that harsh early conditions lead to the development of a body that is less likely to survive at any age, regardless of the future state of the external environment (Wells, 2012). Consequently, it is advantageous to reach maturity early after experiencing early adversity to increase the likelihood of achieving some level of reproduction. What individuals are “predicting” is not the condition of the external environment during their adulthood, but rather the future state of their own body (Nettle et al., 2013). Longitudinal studies in humans have successfully shown a link between environmental harshness during ontogeny with adult coping behavior and reproductive strategies (Mell, 2017; Nettle et al., 2013).

A category of theories that stand in contrast to PAR hypotheses are “developmental constraints” models. These models state that when faced with adversity early in life, the adaptive developmental response is to prioritize immediate survival, even at the cost of impairing other developmental aspects. This type of response prevents immediate death or disability but may lead to negative outcomes later in life. An example of this is the “silver spoon effect,” which describes how exposure to favorable conditions during development can lead to fitness benefits in adulthood. Conversely, exposure to adverse conditions during development can lead to disadvantages later in life (Grafen, 1988). Another example is the “thrifty phenotype hypothesis,” which states that restricted metabolic and behavioral adaptations of malnourished individuals are adaptive as they promote survival in continuously harsh environments (Wells, 2009). Similarly, the mitigation developmental plasticity theory proposes that developing individuals divert energy from expensive morphological developments to maintain essential physiological functions (Smallegange et al., 2019). Compared to the PAR hypotheses that suggest that individuals raised under harsh early-life conditions will fare better in the face of aversive stimuli than those raised in benign conditions, the silver spoon hypothesis predicts that individuals raised under harsh conditions in early-life will exhibit reduced fitness in all environments compared to those raised in benign conditions (Lea et al., 2017).

Currently, neither POLS model formulated within the gene- or development-centric perspective discussed in this study incorporates adaptive developmental hypotheses (predictive-adaptive responses) or developmental constraint models. Selection acts on phenotypes, but because development can alter the trajectory of phenotype development through e.g., maternal and other early life effects, it can bias the direction of evolution (*developmental bias*) (Uller et al., 2018). It is therefore logical to take the development-centric perspective to incorporate developmental hypotheses into POLS models. Milocco & Uller, (2023), for example, developed a modeling framework that represents the organism and environment as a single coupled dynamical system in terms of inputs and outputs, which can be used to model plasticity as a dynamical property that changes in time during ontogeny. Furthermore, Nettle et al., (2013) used a statistical approach and incorporated both internal and external PAR hypotheses into a single probability model, but without life stage structure or a mechanistic underpinning. However, a method grounded in processes can encapsulate the constructive nature of development and plasticity. This approach is often favored over statistical methods because these typically concentrate on a specific point in development and are thus ill-equipped to tackle questions about how a plastic response is influenced by the timing of an environmental shift (Milocco & Uller, 2023).

While links between life history and behavior at the within-population level are increasingly documented, few studies have investigated whether such links translate to among-population and species levels and these studies have found limited transferability. Debecker et al., (2016) reared four closely related species of damselflies in a common garden experiment, measuring four life-history and two behavioral traits. While life-history traits aligned along the expected slow-fast continuum and behavioral traits varied from proactive to reactive, the covariation of life history and behavior depended on the species and the sex, thus supporting the POLS in some but not all species and not across species. Careau et al., (2009) showed a positive correlation between the age of first reproduction and active exploration across 19 muroid species. In the house mouse, one of the species included in (Careau et al., 2009), such a link was also confirmed at the within-population level (Prahb et al., 2023) and Eccard et al., (2022) confirmed a similar link at the within-population level for common voles.

## Outstanding research questions

Considering the relative balance of variation in energy allocation compared to energy acquisition between individuals, and how we expect variation in life history, physiological, and behavioral traits to be linked to either or both processes, is a good first step towards transforming the modern POLS framework into a more predictive framework about how life history, physiological, and behavioral traits vary consistently across populations and species (see Box 1 for a practical guide). In the future, it can be applied to study POLSs within populations (Box 3), but also go beyond POLS by providing clarifications for general life-history research (Duckworth, 2010). This will enable us to tackle significant yet unresolved questions regarding the generation of POLSs and their influence on the ecology and evolution of populations.

For example, the expression of many phenotypes is directly linked to the acquisition and allocation of energy (Biro & Stamps, 2008; Pierce et al., 2024); one prominent example of which is the condition-dependent expression of alternative phenotypes such as described for the bulb mites. Other examples include thermal preferences, whereby individuals with higher thermal preferences tend to be more aggressive, bolder, and active than those operating at lower body temperatures (Goulet et al., 2017). High body temperatures are associated with higher energetic demands, and increased environmental temperatures such as predicted by global warming, affect both behavioral phenotypes as well as energy budgets (Gopal et al., 2023). Such effects on POL-syndromes could be tackled using the energy budget approach proposed here. More generally, such an effort could contribute to our understanding of how the POLS compares to other syndromes and whether there are similar mechanisms underlying different described syndromes? For example, the dispersal syndrome describes how fast types, i.e., individuals with high rates of energy expenditure, boldness, and activity, disperse (Bowler & Benton, 2005; Clobert et al., 2009). Dispersal directly feeds back to population dynamics and influences for example invasion capabilities (Clobert et al., 2009). Also, syndromes often represent negative associations among functional traits (emerging due to underlying trade-offs) (Agrawal, 2020). Can an understanding of the mechanisms generating such negative associations help us to predict niche choice, niche conformance, or niche construction of species, populations, or individuals (Box 2)? Finally, high rates of environmental change typically decrease population sizes (Spooner et al., 2018) and long-lived species with a slow life-history are expected to survive longer at small population sizes and therefore to be buffered against environmental change (O’Grady et al., 2004). Short-lived species are thought to collapse quickly at low population sizes in unfavorable environments. Does the development-centric perspective allow us to draw new conclusions about the influence of environmental variability and life history strategies (Box 1)? The latter would be especially interesting in the context of urbanization (Hahs et al., 2023) to assess to what extent anthropogenic selection pressures drive the evolution of POLS, given their clear impact on life history traits.

## Glossary:

Acquisition: Collection and consumption of resources from the environment
Agency: the ability of an organism to interact with its environment in a purposeful and functional way
Allocation: Division of resources between two or more competing processes within an individual
Development: the process by which organisms grow and develop, from a young juvenile to a reproductive adult
Developmental plasticity: a widespread phenomenon in nature whereby individuals, in response to internal and/or external cues, follow alternative developmental trajectories that anticipate future environmental conditions or mitigate current environmental stressors, ultimately resulting in lasting alterations in phenotype
Development-centric perspective: assumes that natural selection favors developmental systems that tend to construct phenotypes that are successful, relative to other variant systems, at surviving and reproducing
Fast-paced types: individuals that grow fast and large, have high reproductive rates, and have high total fitness. Yet, at high population densities, their maintenance costs will exceed their total amount of assimilated energy and they will be outcompeted by slow-paced types
Fluctuating density-dependent selection: A form of natural selection, wherein the strength and direction of selection on a trait will depend on the density of the local population of a species as environmental conditions fluctuate
Gene-centric perspective: assumes that the evolution of phenotypes results from fitness differences in phenotypes that are specified by genes and genotypes
Multi-trait syndromes: suites of phenotypically and/or genetically integrated traits such as life history, behavior, physiology, and morphology
Pace-of-life-syndrome: comprising suites of correlated life history, physiological and behavioral traits emerging from different solutions to life-history trade-offs
Phenotypic plasticity: the phenomenon that phenotype expression depends on environmental effects, given a particular genetic background
Slow-paced types: avoid impairment at high densities because they have lower maintenance costs, but their growth and reproduction are intrinsically slower than that of fast-paced types, lowering their total fitness.
